# A novel placement method of a calibration‐free pH capsule for continuous wireless measurement of intragastric pH in horses

**DOI:** 10.1111/jvim.17273

**Published:** 2024-12-23

**Authors:** Evelyn Hodgson, Marthe Thirouin, Pranav Narayanan, Tallia‐Rume Romano, Jessica Wise, Stephanie Bond

**Affiliations:** ^1^ School of Veterinary Science, Faculty of Science University of Queensland Gatton, Queensland 4343 Australia

**Keywords:** EGGD, EGUS, equine, ESGD, gastric, ulcers

## Abstract

**Background:**

Current methods to measure intragastric pH in horses have limitations. A wireless capsule has been designed for continuous esophageal pH monitoring in humans.

**Objectives:**

To (1) determine the feasibility and describe the methodology of measuring intragastric pH wirelessly in horses; and (2) determine attachment duration of the capsules.

**Animals:**

Eleven healthy adult horses.

**Methods:**

Capsules were attached to squamous and glandular gastric mucosa under gastroscopic guidance, using suture loops and 1 to 4 hemostasis clips. pH was continuously recorded using a wireless recorder in both fed and fasted states. Gastroscopy was performed daily to assess capsule attachment and any mucosal damage. Data were analyzed using commercially available software. Values are reported as median (interquartile range).

**Results:**

Capsules were successfully placed and data obtained in squamous (n = 11) and glandular (n = 7) regions. The overall duration of squamous capsule attachment was 27 hours (15‐32); 1 clip (n = 4) was 15 hours (11‐20), 2 clips (n = 2) was 20 hours (16‐23), 3 clips (n = 4) was 32 hours (30‐32), and 4 clips (n = 1) was 33 hours. The overall duration of glandular capsule attachment was 10 hours (8‐21); 1 clip (n = 2) was 11 hours (10‐13), 2 clips (n = 2) was 19 hours (14‐23), 3 clips (n = 2) was 7 hours (7‐8), and 4 clips (n = 1) was 158 hours. There was no substantial damage to the gastric mucosa as a consequence of attachment.

**Conclusions and Clinical Importance:**

This novel technique enables the wireless measurement of intragastric pH in horses at known locations under fed and fasted conditions, providing a viable alternative for continuous monitoring in both research and clinical scenarios.

**Abbreviations**
%tpH < 4: percentage of time intragastric pH is less than 4

## 1 INTRODUCTION

Equine gastric ulcer syndrome is the most common disease of the equine stomach,^1^ with a prevalence of 11%‐100% depending on breed and discipline.^2^, ^3^ Monitoring gastric pH is key when evaluating therapeutic interventions, however, current methods have limitations. Precise measurement is important, as acid secretion by the glandular mucosa and fibrous mat formation create intragastric pH stratification with a pH gradient from 6 to 7 at the cardia to 1‐3 in the ventral fundus.^4^, ^5^, ^6^, ^7^, ^8^ Gastric fluid aspiration only provides point‐in‐time measurements^9^, ^10^, ^11^ and often requires sedation and feed withholding which can both affect pH.^12^, ^13^, ^14^, ^15^ Indwelling nasogastric pH probes have been used for 24‐72‐hour monitoring^6^, ^8^, ^15^, ^16^, ^17^, ^18^ and are well tolerated, but the electrode location is unknown with potential for displacement.^19^ Gastric cannulation^5^, ^20^, ^21^, ^22^ and percutaneous endoscopic gastrotomy tubes^23^, ^24^ have also been described; while these techniques enable continuous measurement in fed horses with known electrode location, they are invasive and only suitable in the research context. A free wireless capsule successfully measured continuous pH non‐invasively in ponies, however, data transmission was insufficient in adult horses.^25^ Furthermore, the exact location of this capsule cannot be determined.

In people with gastroesophageal reflux disease, a wireless capsule is attached to the distal esophagus via suction for 24‐96‐hour pH measurement.^26^, ^27^ Several studies have measured intragastric pH using this method in humans,^28^, ^29^ monkeys,^30^ dogs,^31^ and cats,^32^ and a novel method used hemostasis clips to attach the capsule to the gastric mucosa in people.^33^, ^34^

The objectives of the present study were to: (a) investigate the feasibility of a novel placement method using hemostasis clips to attach a wireless capsule to the squamous and glandular mucosa in horses for continuous measurement of intragastric pH; (b) describe an optimized methodology; and (c) determine attachment duration. Our hypothesis was that the capsules could be attached to the squamous and glandular gastric mucosa using hemostasis clips and obtain continuous wireless pH readings in the horse.

## 2 MATERIALS AND METHODS

### 2.1 Study cohort and design

Eleven healthy adult mares (4 Standardbreds and 7 Australian Stock Horses) from the University of Queensland Equine Unit, aged 5‐26 years and weighing 421‐515 kg, were enrolled in a prospective pilot study. Complete physical examinations were performed to exclude the presence of clinical disease. Horses were housed in pairs in adjacent dirt yards with a shelter, and fed lucerne hay ad libitum with free access to fresh water. The research protocol was approved by the University of Queensland Animal Ethics Committee (2023/AE000713).

### 2.2 Capsule preparation

A wireless, calibration‐free pH capsule (6 × 5.5 × 25 mm; Bravo Calibration‐free Delivery Device Capsules FGS‐0635, Medtronic, Shoreview, Minnesota) was used. The capsules were removed from the manufacturer's delivery system and attached to the gastric mucosa using 1 to 4 hemostasis clips (HX‐202UR 2300 mm, Olympus Medical Systems Corporation, Tokyo, Japan). An iterative process was used to develop the optimized methodology which is described below.

**FIGURE 1:**
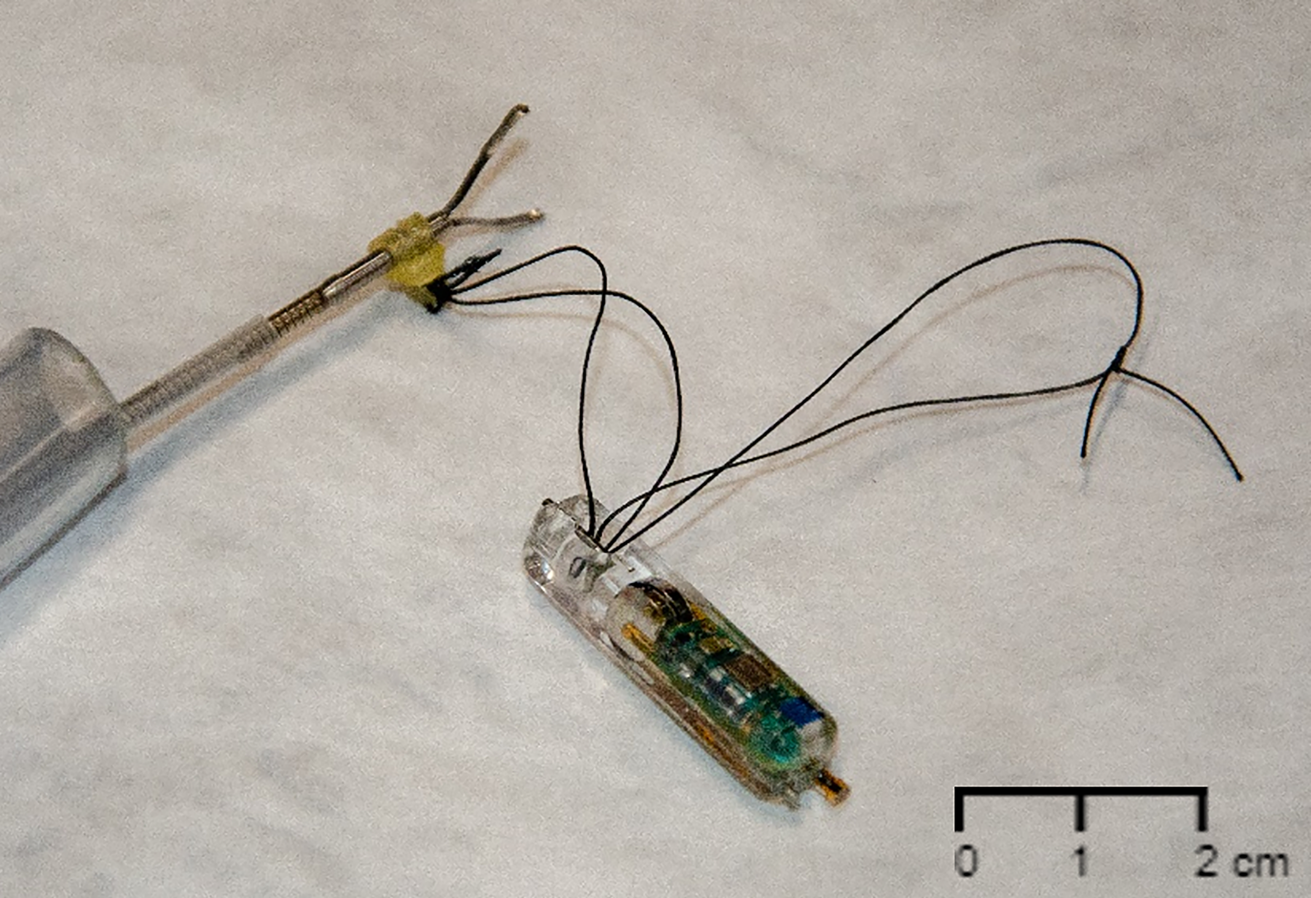
Calibration‐free pH capsule attached to the hemostasis clip via the suture loops and orthodontic elastic.

The capsule was attached to the first clip via an orthodontic elastic (Motion 3D Force 1 Elastic 6oz 1/4, Henry Schein Orthodontics, Carlsbad, California) connected to a loop of silk suture (Ethicon 3‐0, Johnson & Johnson Medical Pty Ltd, North Ryde, NSW, Australia). First, the elastic was passed around the clip and looped through itself, then secured with cyanoacrylate glue. Two 4 cm diameter suture loops were made. One suture loop was connected to the elastic via a loop‐to‐loop knot connection. Both suture loops were then attached to the capsule by placing them into the capsule suction well. The capsule delivery system was deployed, locking a trocar in place which secured the suture loops in the well, and releasing the capsule from the delivery system. The result was a capsule with 2 suture loops; 1 loop attached the capsule to the clip via the orthodontic elastic, and 1 loop enabled attachment with further hemostasis clips once in the stomach (Figure 1).

This modified clip‐capsule device was advanced through a 2150 mm overtube (10 mm internal diameter, Pope Products, Kilkenny, SA, Australia). The capsule was paired to the wireless recorder (Bravo Calibration‐free Reflux Recorder FGS‐0634, Medtronic, Shoreview, Minnesota).

### 2.3 Gastroscopy

Before gastroscopy and capsule placement, feed was withheld for 16 hours and water for 2 hours. Horses were sedated with 0.01 mg/kg detomidine hydrochloride IV (Sedator, Randlab Pty Ltd, Revesby, NSW, Australia) and 0.01 mg/kg butorphanol tartrate IV (Butorphanol, Randlab Pty Ltd, Revesby, NSW, Australia). Selected horses also received 0.02‐0.04 mg/kg acepromazine IV (Acepril‐10, TROY Laboratories Pty Ltd, Glendenning, NSW, Australia) 30 minutes prior.

Gastroscopy was performed using a 3‐m endoscope (Olympus Medical Systems Corporation, Tokyo, Japan). The presence of squamous or glandular ulceration was assessed and graded^1^; exclusion criteria comprised grade 4 equine squamous gastric disease^35^ or severe equine glandular gastric disease.^1^

### 2.4 Capsule placement

The endoscope was retracted to the pharynx and the overtube containing the clip‐capsule device was passed through the contralateral nostril and into the esophagus under endoscopic guidance. The overtube and endoscope were advanced simultaneously into the stomach.

**FIGURE 2:**
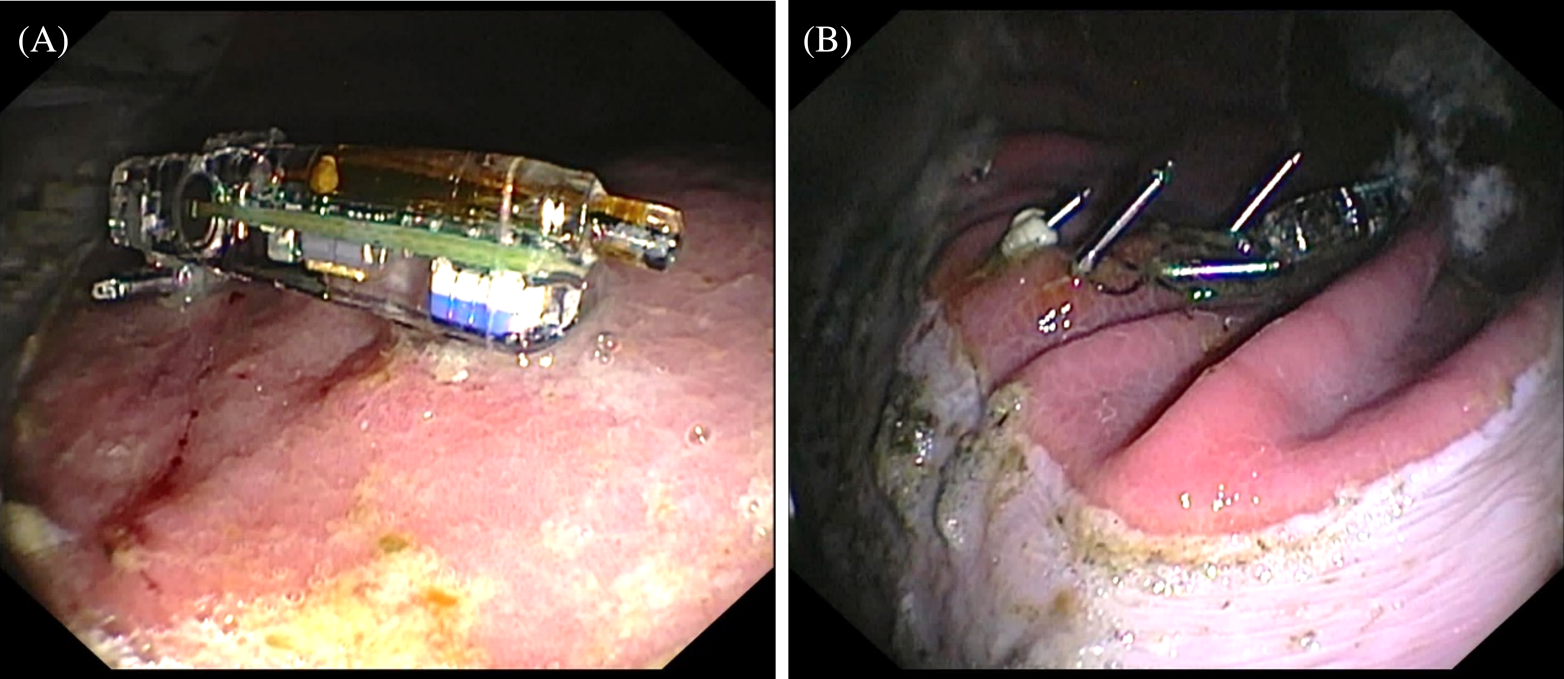
Gastroscopic images showing a capsule attached to the glandular mucosa ventral to the margo plicatus with 1 (A) and 4 (B) hemostasis clips.

The overtube was maneuvered first to the glandular mucosa ventral to the margo plicatus at the greater curvature and the clip‐capsule device was advanced beyond the overtube. Rat tooth alligator forceps (Model 7904 3500 mm, TeleMed Systems Inc, Hudson, Massachusetts) were used to grasp the clip and aid orientation of the capsule when necessary. Mucosal tissue was grasped with the clip, adequate tissue attachment was visually confirmed, and the clip was closed and deployed (Figure 2). An additional 1 to 3 clips were threaded through the overtube and used to further secure the suture loops, and thus the capsule, to the mucosa. The overtube was then removed.

**FIGURE 3:**
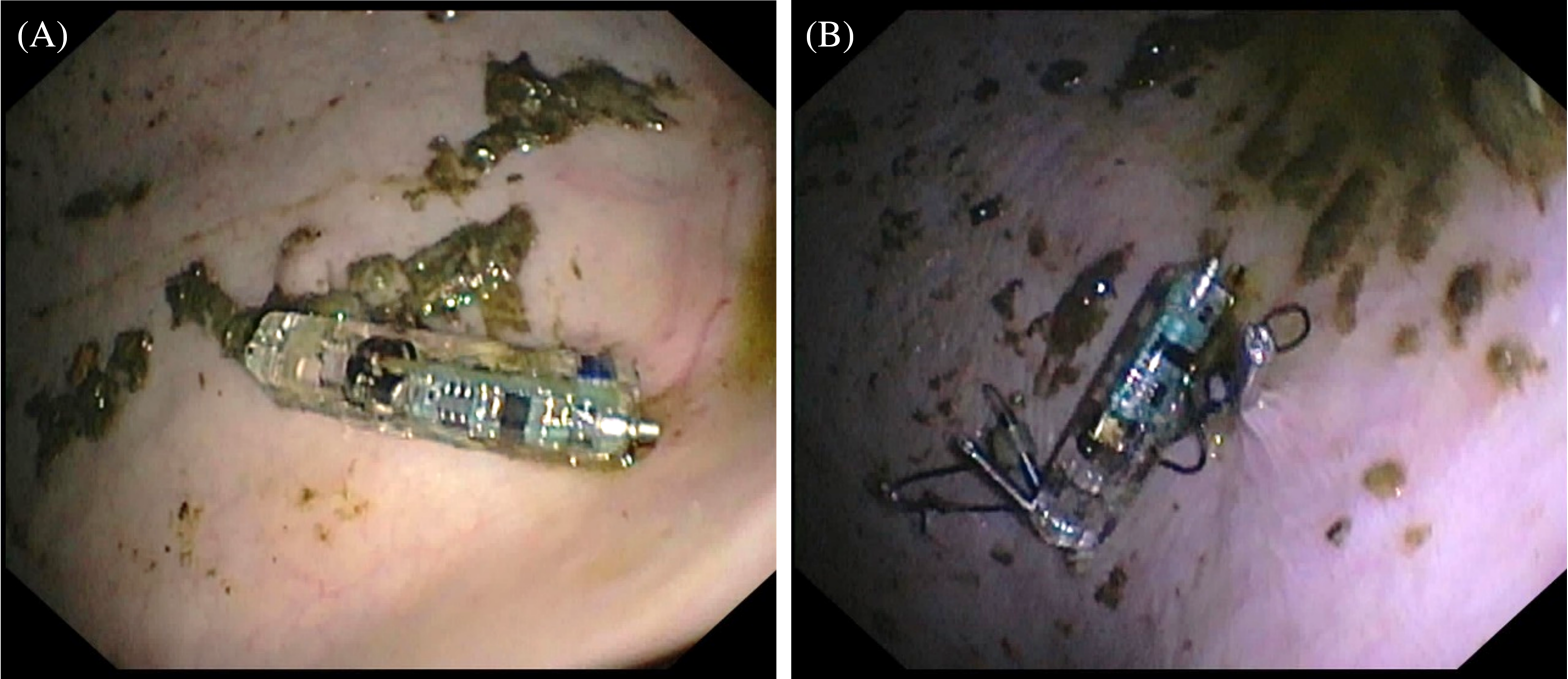
Gastroscopic images showing a capsule attached to the squamous mucosa dorsal to the margo plicatus with 1 (A) and 4 (B) hemostasis clips.

The endoscope was retracted to the pharynx, a second overtube containing a clip‐capsule device was passed through the contralateral nostril, and the procedure was repeated at the squamous mucosa dorsal to the margo plicatus at the greater curvature (Figure 3).

If extensive irritation of the pharyngeal or esophageal mucosa occurred, or procedural time exceeded 30 minutes, 4.4 mg/kg phenylbutazone was administered IV (Platinum Bute, Randlab Pty Ltd, Revesby, NSW, Australia). Ad libitum feed and water were reintroduced once sedative effects subsided.

### 2.5 pH recording

After capsule placement, connection to the recorder was confirmed. The recorder was secured to the horse using a surcingle (Televet Electrode Support, Kruuse, Langeskov, Denmark) behind the point of the left elbow for proximity to the capsules and maximal protection.

The recorder collected pH data via telemetry every 6 seconds. In addition, the instantaneous pH was manually recorded hourly for 12 hours each day. Intragastric pH data were recorded continuously until endoscopic confirmation of capsule detachment. Recording duration was limited to 96 hours, so if capsules were still attached, the data were uploaded and a new recording started.

### 2.6 Monitoring

Horses were monitored for adverse effects of capsule attachment or repeated gastroscopy, sedation and withholding of food. Full physical examination was performed daily before sedation and gastroscopy. Demeanor, heart rate and respiratory rate were monitored hourly for 12 hours after each procedure. Fecal output and food and water consumption were monitored twice daily.

### 2.7 Follow‐up gastroscopy

Gastroscopy was repeated every 24 hours to determine whether the capsules had detached. Feed was withheld for 12 hours prior, but water was not. Horses were sedated with 0.01 mg/kg detomidine IV and gastroscopy was performed as per Section 2.3. The mucosa was examined for any consequences of attachment and development or worsening of gastric ulceration.

### 2.8 Data analysis

**FIGURE 4:**
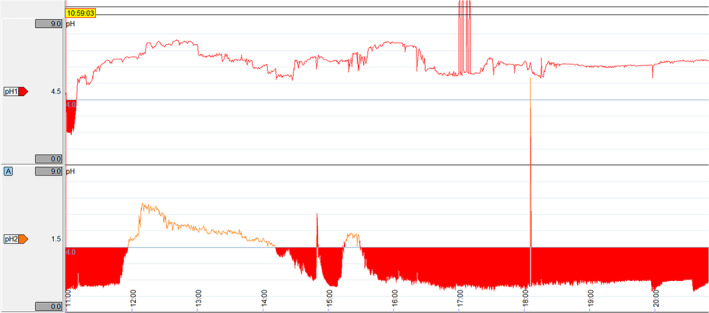
Example of a graph of pH over time for squamous (top) and glandular (bottom) capsules generated by the software. Time (24‐hour clock) is on the x‐axis and pH is on the y‐axis. pH <4 is highlighted by red shading. The sharp vertical lines that extend beyond the y‐axis represent artifacts because of temporary transmission failure.

Following confirmation of capsule detachment, or every 96 hours, pH data were uploaded onto a computer using commercially available software (Reflux Software Version 6.1, Medtronic, Shoreview, Minnesota). Graphs of pH over time were generated by the software (Figure 4) and visually analyzed to determine the point of detachment and calculate attachment duration. The percentage of time the pH was less than 4 (%tpH < 4) was calculated directly. Raw data were then exported as .txt files and imported into commercially available software (Microsoft Excel v16.0, Microsoft, Redmond, Washington). Values above 14 were interpreted as artifacts and removed. Mean pH and median hourly pH were calculated for each capsule. For continuous data, a Shapiro‐Wilk test was performed to assess data distribution. Descriptive statistics were reported as mean ± standard deviation (SD) for normally distributed data, median and interquartile range (IQR) for non‐normally distributed data, and frequencies and percentages for categorical data. The mean %tpH < 4 and the mean of the mean pH were then calculated for all squamous and glandular capsules. Independent *t‐*tests were used to determine whether there was an effect of location on mean pH and %tpH < 4. A 2‐way ANOVA was used to test if there was an effect of location or number of clips on the attachment duration, as well as interaction effects between location and number of clips. Only 1 clip and 3 clips were compared as there were insufficient data points for 2 and 4 clips.

## 3 RESULTS

### 3.1 Capsule attachment

A total of 11 squamous and 7 glandular capsules were attached, and pH measurement was successful in all capsules.

**FIGURE 5:**
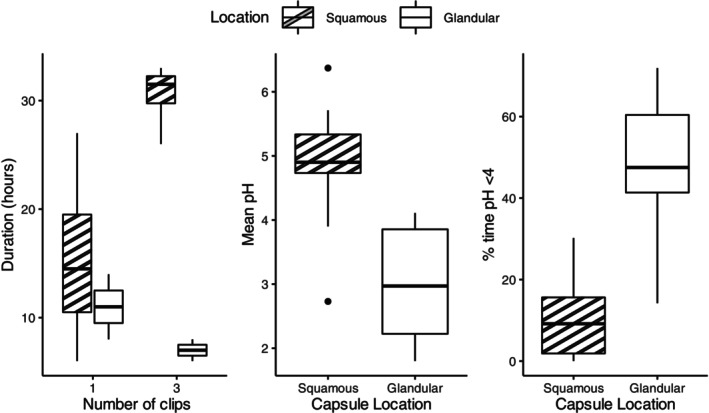
Duration of attachment of capsules with 1 hemostasis clip and 3 hemostasis clips at squamous and glandular locations (A), mean pH throughout duration of attachment at squamous and glandular locations (B) and the percentage of time the pH was less than 4 (%tpH < 4) at squamous and glandular locations (C). The median is marked by the central horizontal line, the box spans the interquartile range, and the whiskers span the upper and lower limits. Outliers are indicated by circles.

The overall range of attachment duration was 6 to 158 hours, with ≥24 hours in 53% of capsules. Attachment duration was significantly longer for squamous capsules (*P* = .005), with a median of 27 hours (15‐32) for squamous capsules, and 10 hours (8‐21) for glandular capsules. Attachment duration increased with number of clips (Table 1), with a significant difference between 1 and 3 clips (*P* = .04; Figure 5A). Location increased duration significantly more if 3 clips were used than if 1 clip was used (*P* = .03).

**TABLE 1:** Median (IQR) duration of attachment of capsules by location and number of hemostasis clips.

Number of clips	Squamous				Glandular			
	1 clip	2 clips	3 clips	4 clips	1 clip	2 clips	3 clips	4 clips
Number (n)	4	2	4	1	2	2	2	1
Attachment duration (h)	15 (11‐20)	20 (16‐23)	32 (30‐32)	33	11 (10‐13)	19 (14‐23)	7 (7‐8)	158

Detachment and passage into the small intestine was suspected with a rapid and sustained increase in pH to ≥8 and/or signal loss, and consistently confirmed via gastroscopy the following morning (6‐20 hours later).

### 3.2 pH recording

Intermittent data transmission failures were observed with a mean data capture rate of 60.7 ± 31.3%. Because of a software malfunction, data from 1 squamous capsule could not be exported and was excluded from pH data analysis.

**FIGURE 6:**
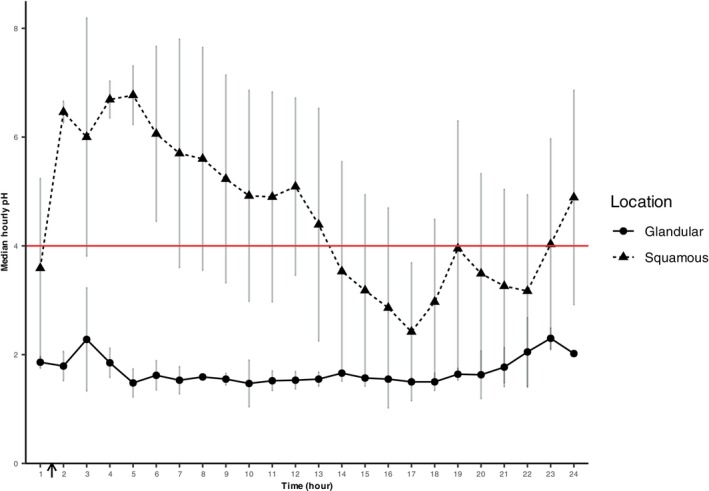
Median (individual) hourly pH at squamous and glandular locations over the first 24 hours after attachment for all capsules with ≥24 hours attachment and pH data (n = 8). Data are shown as mean ± SD. Feed was reintroduced between hours 1 and 2 (black arrow). Feed was withheld overnight from 8 pm and gastroscopy was performed at 8 am the following day, corresponding to periods starting at hours 8‐11 and ending at hours 20‐23.

The mean of the mean squamous pH was 4.9 ± 1.0, with a mean %tpH < 4 of 9.4 ± 9.9%. The mean of the mean glandular pH was 3.0 ± 1.0, with a mean %tpH < 4 of 48.2 ± 19.2%. A significant difference was found between squamous and glandular locations for both mean pH (*P* = .002; Figure 5B) and %tpH < 4 (*P* = .001; Figure 5C). A transient post‐prandial increase and diurnal variations in squamous pH were observed, whereas glandular pH remained stable (Figure 6).

### 3.3 Gastroscopy

Initial gastroscopy identified grade 2 squamous ulceration in 2 horses, which did not worsen upon re‐examination. On day 5, horse 10 developed grade 3 squamous ulceration distant to the clip attachment sites. Consequently, omeprazole treatment was initiated (1 mg/kg PO SID; Gastropell Daily, Randlab Pty Ltd, Revesby, NSW, Australia). Gastroscopy after 14 days of treatment showed complete healing. pH data were excluded from pH analysis after initiation of treatment.

**FIGURE 7:**
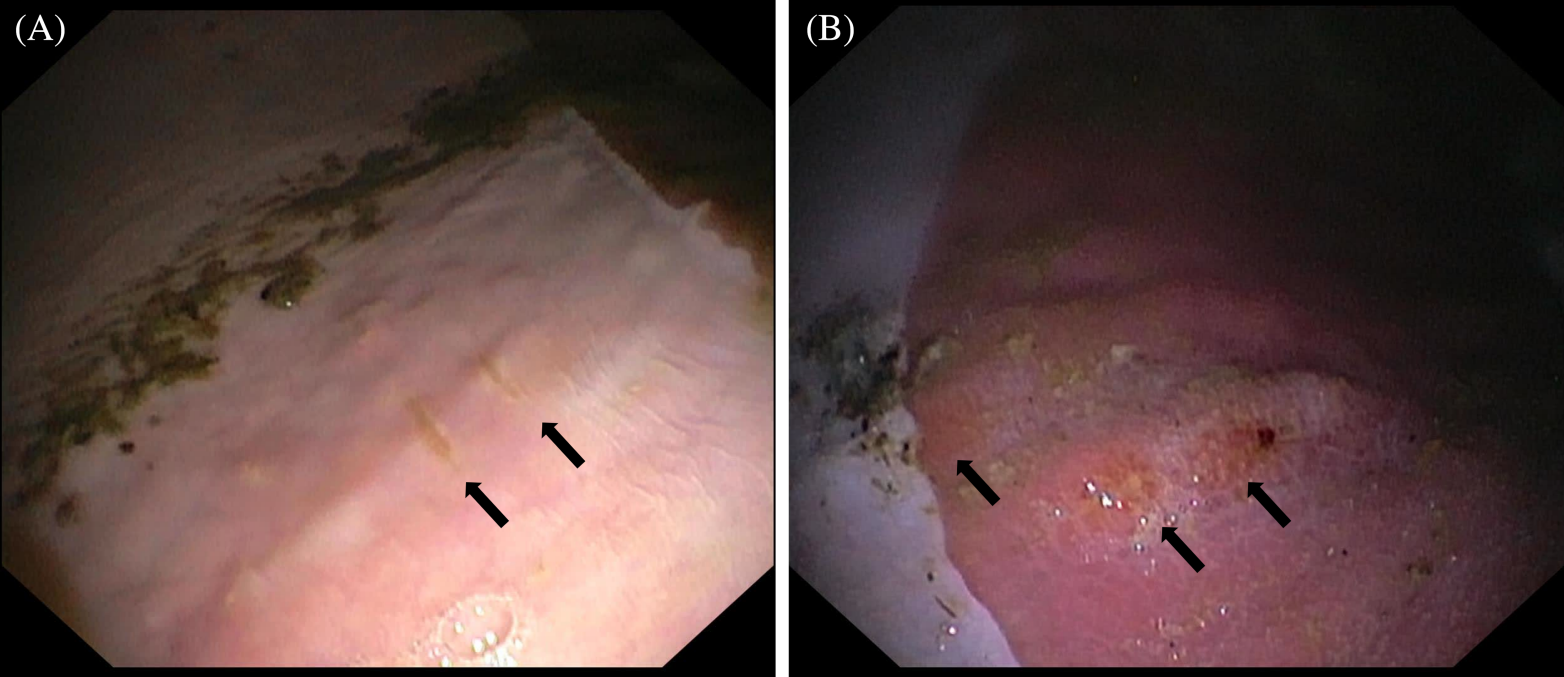
Gastroscopic images showing superficial erosions on the squamous mucosa (A) and hyperemia of the glandular mucosa (B; black arrows) after detachment of the hemostasis clips.

Mild focal lesions were identified at the attachment sites in 6 horses, including mucosal erosions (Figure 7A) and hyperemia (Figure 7B).

### 3.4 Monitoring

The capsules and clip attachments were well tolerated by all horses with no complications or adverse effects. Elimination of the capsule was not confirmed.

## 4 DISCUSSION

Capsules were successfully attached to the squamous and glandular gastric mucosa of horses with hemostasis clips, enabling continuous wireless intragastric pH recordings. While the attachment duration was variable, 53% of capsules remained attached for ≥24 hours and retention improved with additional clips and technique refinement. Although some groups had insufficient sample sizes for statistical analysis, we demonstrated that the use of more clips substantially increased the attachment duration. Attachment duration was shorter than equivalent human studies, where 80% of capsules remained attached for 48 hours with 1 hemostasis clip,^33^ and 71% remained attached for 16 days with 4 clips.^34^ This disparity might be because of species differences in diet,^36^ gastric motility,^36^, ^37^, ^38^ or variation in type^1^, ^39^ and thickness of gastric mucosa.^40^, ^41^ In the present study, glandular capsules had a shorter attachment duration, which might be explained by local motility dynamics. Interestingly, in horse 10, the glandular capsule remained attached for 158 hours with 4 clips, far exceeding the overall average. Though a longer and more consistent attachment is desirable, premature detachment also occurs in humans^26^, ^33^, ^42^ and small animals.^31^ Recording duration in horses with nasogastric pH probes is also variable because of premature removal, breakage or failure of the probe or data logger,^16^, ^18^, ^43^, ^44^ with percentages of ≥24‐hour recordings reported as low as 33%.^18^

Although high rates of captured data (up to 99.7%) were achievable, large inter‐individual variability was observed, with considerable intermittent transmission failures. Missing data are a reported complication of wireless pH capsule studies in humans^26^, ^42^, ^45^ and animals,^31^, ^32^ albeit at a much lower rate, because of poor signal reception, insufficient electrode contact, or electrical faults.^45^ The body mass of the horse is likely to contribute to inferior transmission rates; poor connection has also been observed in horses with other intestinal capsules requiring real‐time transmission.^25^, ^46^ Concurrent aspiration of gastric fluid has been used to mitigate missing data in nasogastric probe studies,^16^, ^17^ but might not be suitable in conjunction with this method because of potential for interference and capsule detachment. An advantage of this capsule is the calibration‐free feature which automatically corrects for pH drift, thereby avoiding the exclusion of data because of pH drift or failure of calibration,^16^, ^44^ as well as reducing labor intensity.

Capsule attachment was well tolerated by all horses with no adverse effects. The most commonly reported side effects with mucosal suction in humans are foreign body sensation and mild to severe chest pain,^26^, ^29^ which have not been reported with the clipping method.^33^, ^34^ Although nasogastric pH probes are largely well tolerated in horses, some studies describe reduced eating behavior,^6^ and they cause significant discomfort in humans leading to diet and activity restriction.^45^ The development of squamous ulceration in horse 10 was attributed to daily withholding of feed^12^ and not capsule attachment, as it occurred distant to the attachment sites. Although pH data were excluded from analysis following omeprazole treatment, the horse displayed an expected increase in pH and decrease in %tpH < 4.

A significant effect of location on pH and %tpH < 4 was identified, as well as temporal patterns, which parallel the findings of Husted et al.^6^ Although our horses were fasted nightly, the observed nocturnal decrease in pH has been shown to occur equally in fed and fasted horses.^12^ These findings highlight the importance of known electrode location when collecting and interpreting intragastric pH data.

Detachment of the capsule could be reliably identified by a rapid and sustained increase in pH to ≥8 and/or signal loss, which was corroborated by gastroscopy. Similar pH profiles have been reported in wireless pH capsule studies in other species,^30^, ^31^, ^47^ as well as free wireless capsules in horses.^25^ It is important to visually inspect the pH graphs and confirm a sustained increase, as short bursts of increased pH may represent enterogastric reflux or saliva.^36^ If detachment is suspected, gastroscopy is recommended for confirmation.

This novel technique enabled the continuous wireless measurement of intragastric pH at known locations in the equine stomach under fed and fasted conditions, offering a valuable alternative model for pharmacodynamic studies and potential for clinical use. It has clear advantages compared to nasogastric pH probes, with their unknown electrode location and propensity for displacement,^19^, ^45^ and to invasive interventions such as indwelling percutaneous gastrotomy tubes or gastric cannulation.^20^, ^24^

## CONFLICT OF INTEREST DECLARATION

Authors declare no conflict of interest.

## OFF‐LABEL ANTIMICROBIAL DECLARATION

Authors declare no off‐label use of antimicrobials.

## INSTITUTIONAL ANIMAL CARE AND USE COMMITTEE (IACUC) OR OTHER APPROVAL DECLARATION

Approved by the University of Queensland Animal Ethics Committee (2023/AE000713).

## HUMAN ETHICS APPROVAL DECLARATION

Authors declare human ethics approval was not needed for this study.
